# Increased levels of low-density lipoprotein cholesterol within the normal range as a risk factor for nonalcoholic fatty liver disease

**DOI:** 10.18632/oncotarget.6799

**Published:** 2015-12-30

**Authors:** Dan-Qin Sun, Wen-Yue Liu, Sheng-Jie Wu, Gui-Qi Zhu, Martin Braddock, Dong-Chu Zhang, Ke-Qing Shi, Dan Song, Ming-Hua Zheng

**Affiliations:** ^1^ Department of Nephrology, Affiliated Wuxi Second Hospital, Nanjing Medical University, Wuxi 214002, China; ^2^ Department of Endocrinology, the First Affiliated Hospital of Wenzhou Medical University, Wenzhou 325000, China; ^3^ Department of Cardiovascular Medicine, the Heart Center, the First Affiliated Hospital of Wenzhou Medical University, Wenzhou 325000, China; ^4^ Department of Infection and Liver Diseases, Liver Research Center, the First Affiliated Hospital of Wenzhou Medical University, Wenzhou 325000, China; ^5^ School of the First Clinical Medical Sciences, Wenzhou Medical University, Wenzhou 325000, China; ^6^ Global Medicines Development, AstraZeneca R&D, Alderley Park, United Kingdom; ^7^ Wenzhou Medical Center, Wenzhou People's Hospital, Wenzhou 325000, China; ^8^ Institute of Hepatology, Wenzhou Medical University, Wenzhou 325000, China

**Keywords:** low-density lipoprotein cholesterol, non-alcoholic fatty liver disease, risk factor

## Abstract

**Objectives:**

Dyslipidemia exists within the setting of NAFLD and the relationship of a normal level of low-density lipoprotein cholesterol (LDL-c) with NAFLD is largely unknown. This large population-based study aimed to investigate the association between LDL-c levels within the normal range and the incidence of NAFLD.

**Methods:**

A total of 60527 subjects from 2 medical centers who had undergone liver ultrasonography were initially enrolled into this study. NAFLD was defined by ultrasonographic detection of steatosis in the absence of other liver disease. Subjects were divided into 4 groups (Q1 to Q4) by normal LDL-c quartiles : Q1: ≤ 2.00, Q2: 2.10-2.35, Q3: 2.36-2.68 and Q4: 2.69-3.12 mmol/L. The odds ratios (OR), hazard ratio (HR) and 95% confidence intervals (CIs) for NAFLD were calculated across each quartile of LDL-c, using the Q1 as reference.

**Results:**

The prevalence rates of NAFLD in a cross-sectional population from Q1 to Q4 were 19.34%, 25.86%, 35.65% and 42.08%, respectively. The OR for NAFLD in the cross-sectional population were 1.31 (95% CI 1.14-1.54), 1.73 (95% CI 1.46-2.04), and 1.82 (95% CI 1.49-2.23), respectively, after adjusting for known confounding variables. The HR for NAFLD in the longitudinal population were 1.23 (95% CI 1.12-1.35), 1.57 (95% CI 1.44-1.72) and 2.02 (95% CI 1.86-2.21), compared with Q1. Subjects with higher LDL-c level within the normal range had an increased cumulative incidence rate of NAFLD.

**Conclusions:**

Increased levels of LDL-c within the normal range may play a significant role in the prevalence and incidence of NAFLD, independent of other confounding factors.

## INTRODUCTION

Non-alcoholic fatty liver disease (NAFLD) is a major, worldwide public health problem and is defined as the accumulation of excessive fat in the liver in the absence of quantities of alcohol and any secondary cause [1]. It comprises a spectrum of pathologic conditions including simple nonalcoholic steatosis, nonalcoholic steatohepatitis (NASH) and hepatic cirrhosis [2]. The prevalence of NAFLD is 20% to 30% of the general population in the western world and 15% to 30% in Asian countries [3]. Recently, increasing attention has been paid to the clinical association of NAFLD and cardiovascular disease (CVD) [4–5]. It is widely accepted that NAFLD plays an important role in subclinical atherosclerosis as well as overt cardiovascular events [6–7]. CVD is the leading cause of death in patients with NAFLD [8], and CVD may also lead to the development of liver disease and increase the morbidity and mortality burden of patients with NAFLD [6]. The classic common risk factors for NAFLD and CVD are age and gender, physical inactivity, type 2 diabetes mellitus, obesity, hypertension and hyperlipidemia [5,9].

Lipoprotein metabolism has two main consequences for the function of lipoproteins. First, lipoproteins are delivered as cholesterol and triglyceride molecules from the liver and intestine to muscle and fat tissue by chylomicrons and very low density lipoprotein (VLDL) particles. Secondly excess cholesterol from extra-hepatic tissues is transported to the liver for elimination via the bile by high density lipoprotein (HDL) particles [10]. Dyslipidemia exists in the setting of NAFLD, which results in hypertriglyceridemia, reductions in high density lipoprotein cholesterol (HDL-c) and an increase in the size of VLDL [11]. These changes are typically accompanied by increased concentrations of atherogenic low density lipoprotein cholesterol (LDL-c), even when adjusted for metabolic risk factors [12]. Several studies have shown that some risk markers, including C-reactive protein, LDL-c, interleukin (IL)-6 and plasminogen activator inhibitor-1 are associated with NAFLD and the development of NASH [13–14]. However, it is unclear whether an elevated LDL-c level is a risk factor for NAFLD and there is no data examining the association between LDL-c within the normal range and NAFLD. Therefore, identifying potential risk factors is essential for the prevention of NAFLD.

In this study, we aimed to determine the association between normal LDL-c within the normal range and the risk of developing NAFLD from a large general cross-sectional population. A further investigation was performed in a prospective longitudinal population.

## RESULTS

### Characteristics of study subjects

**Figure 1:**
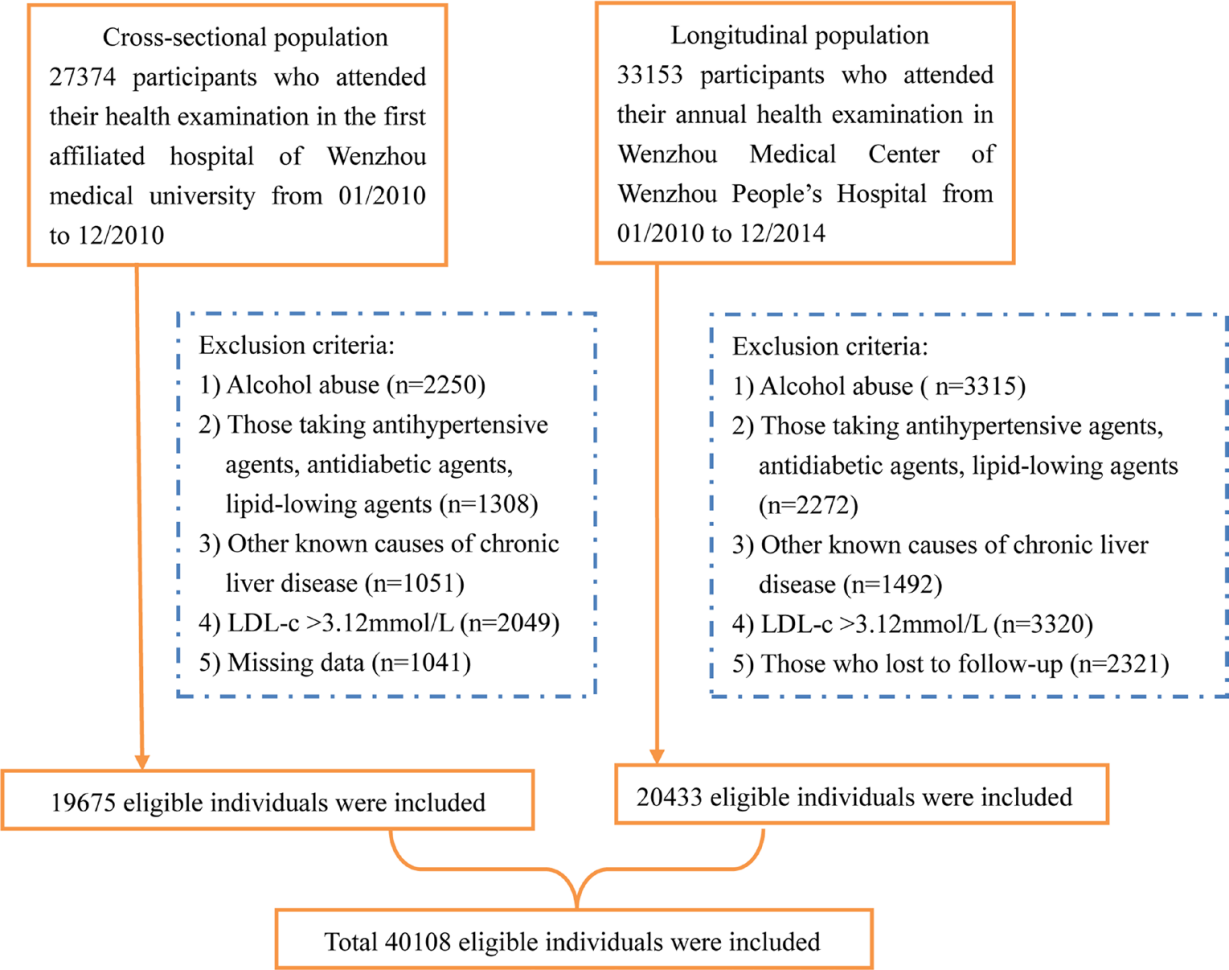
Study flow diagram A total of 27374 participants were enrolled initially, while 19675 participants were included in the cross-sectional population. A total of 33153 participants were enrolled initially, while 20433 participants were included in the longitudinal population.

A total of 60527 subjects were initially enrolled into the study, of which 40108 subjects remained. In the cross-sectional population, 19675 subjects who had undergone liver ultrasonography were enrolled. Patients with incomplete laboratory or clinical data were excluded from analysis (*n* = 1041). In addition, we excluded patients with a history of alcohol abuse, LDL-c > 3.12mmol/L, viral hepatitis B or C and drug induced liver injury. As a result, 5689 subjects met our criteria and were included in the cross-sectional analysis (Figure 1). Table 1 shows the characteristics of study subjects according to their quartile measurements of normal LDL-c range. The prevalence rates of NAFLD gradually increased as the LDL-c level increased. BMI, SBP, DBP, FPG, ALT, AST, BUN, Cr, TC, TG, UA were significantly higher, while HDL-c was lower, among subjects with higher LDL-c levels. In our longitudinal population, 33153 participants attended their annual health examination in 2 medical centers. Patients with incomplete liver ultrasonography were excluded (*n* = 487) in the 5-year follow-up examination. In addition, 1834 subjects who had incomplete laboratory data or lost to follow-up were therefore excluded. Finally, 20433 subjects were included, which completed the 5-year follow-up examination. The baseline characteristics of subjects in longitudinal population are shown in Table 2. A similar change in the measured clinical characteristics was observed with the cross-sectional population.

**Table 1:** Baseline Characteristics of Cross-sectional Population, Stratified by Quartiles of LDL-c

Quartiles of LDL-c (mmol/L) in Cross-sectional Population					
Characteristics	Q1 (<2.0)	Q2 (2.0-2.35)	Q3 (2.36-2.68)	Q4 (2.68-3.12)	*P*
N	4926	3372	5091	4869	
M(F)	2500 (2426)	1984 (1388)	3243 (1848)	3352 (1517)	
NAFLD, N (%)	953 (19.34%)	872 (25.86%)	1815 (35.65%)	2049 (42.08%)	<0.001
Age, y	40.88 ± 12.10	42.15 ± 11.67	44.18 ± 11.56	45.54 ± 11.28	<0.001
BMI, kg/m^2^	22.12 ± 3.61	22.72 ± 3.43	23.49 ± 5.03	23.49 ± 3.46	<0.001
SBP, mmHg	121.54 ± 17.47	123.04 ± 16.85	125.76 ± 17.00	127.51 ± 16.90	<0.001
DBP, mmHg	74.71 ± 11.11	75.63 ± 11.11	77.81 ± 10.93	79.18 ± 11.00	<0.001
FPG, mmol/L	5.09 ± 1.00	5.14 ± 1.03	5.22 ± 1.01	5.32 ± 1.22	<0.001
ALB,U/L	44.48 ± 3.00	44.48 ± 3.01	44.59 ± 2.87	44.58 ± 2.86	<0.001
ALT, U/L	22.51 ± 18.70	24.13 ± 20.71	26.00 ± 19.79	28.80 ± 22.11	<0.001
AST, U/L	24.49 ± 15.39	24.78 ± 13.91	25.25 ± 11.69	26.39 ± 11.90	<0.001
BUN, mmol/L	4.40 ± 1.29	4.57 ± 1.23	4.70 ± 1.22	4.79 ± 1.23	<0.001
Cr, μmol/L	77.51 ± 21.53	78.57 ± 17.56	80.10 ± 18.34	82.00 ± 17.63	<0.001
TC, mmol/L	3.94 ± 0.66	4.46 ± 0.51	4.95 ± 0.50	5.42 ± 0.53	<0.001
TG, mmol/L	1.53 ± 1.59	1.62 ± 1.42	1.79 ± 1.32	1.97 ± 1.46	<0.001
HDL-c, mmol/L	1.42 ± 0.42	1.90 ± 0.36	1.39 ± 0.34	1.39 ± 0.33	<0.001
UA, μmol/L	291.60 ± 95.21	306.07 ± 95.42	320.01 ± 96.40	333.10 ± 96.88	<0.001

Abbreviations: NAFLD = nonalcoholic fatty liver disease, ALB = albumin, ALT = alanine aminotransferase, AST = aspartate aminotransferase, BMI = body mass index, BUN =blood urea nitrogen, Cr = creatinine, SBP = systolic blood pressure, DBP = diastolic blood pressure, FPG = fasting plasma glucose, HDL-c = high-density lipoprotein cholesterol, LDL-c = low-density lipoprotein cholesterol, UA = uric acid, TC = total cholesterol, TG = triglyceride.

**Table 2:** Baseline Characteristics of Longitudinal Population, Stratified by Quartiles of LDL-c

Quartiles of LDL-c in Longitudinal Population					
Characteristics	Q1 (< 2.0)	Q2 (2.0-2.35)	Q3 (2.36-2.68)	Q4 (2.68-3.12)	*P*
N	5739	5087	5116	4491	
M (F)	2971 (2768)	2686 (2401)	2783 (2333)	2431 (2060)	
NAFLD, N (%)	837 (14.58%)	945 (18.58%)	1183 (23.12%)	1288 (28.68%)	P<0.001
Age, y	42.52 ± 14.82	43.15 ± 14.96	43.72 ± 15.02	44.06 ± 15.24	P<0.001
BMI, kg/m^2^	21.52 ± 2.76	21.97 ± 2.63	22.51 ± 2.68	22.81 ± 2.62	P<0.001
SBP, mmHg	119.74 ± 17.45	121.51 ± 16.75	123.51 ± 16.97	125.93 ± 16.89	P<0.001
DBP, mmHg	71.90 ± 10.53	73.34 ± 10.53	74.95 ± 10.86	75.82 ± 10.56	P<0.001
FPG, mmol/L	5.13 ± 0.86	5.15 ± 0.75	5.22 ± 0.83	5.19 ± 0.84	P<0.001
ALB, g/L	44.17 ± 2.80	44.45 ± 2.68	44.48 ± 2.73	44.36 ± 2.73	P<0.001
ALT, U/L	19.79 ± 19.02	20.94 ± 17.90	21.67 ± 15.68	22.94 ± 16.83	P<0.001
AST, U/L	23.05 ± 10.47	23.43 ± 12.21	23.41 ± 9.69	24.07 ± 10.04	P<0.001
BUN, mmol/L	4.50 ± 1.46	4.55 ± 1.41	4.62 ± 1.32	4.74 ± 1.35	P<0.001
Cr, μmol/L	78.15 ± 30.16	78.86 ± 26.43	80.67 ± 22.16	82.71 ± 22.45	P<0.001
TC, mmol/L	3.89 ± 0.53	4.50 ± 0.44	4.93 ± 0.47	5.42 ± 0.48	P<0.001
TG, mmol/L	1.21 ± 0.98	1.33 ± 0.97	1.47 ± 0.34	1.55 ± 0.86	P<0.001
HDL-c, mmol/L	1.45 ± 0.39	1.44 ± 0.35	1.43 ± 0.36	1.44 ± 0.34	P<0.001
UA, μmol/L	273.13 ± 89.20	284.31 ± 87.12	293.95 ± 87.76	301.62 ± 86.63	P<0.001

Abbreviations: NAFLD = nonalcoholic fatty liver disease, ALB = albumin, ALT = alanine aminotransferase, AST = aspartate aminotransferase, BMI = body mass index, BUN =blood urea nitrogen, Cr = creatinine, SBP = systolic blood pressure, DBP = diastolic blood pressure, FPG = fasting plasma glucose, HDL-c = high-density lipoprotein cholesterol, LDL-c = low-density lipoprotein cholesterol, UA = uric acid, TC = total cholesterol, TG = triglyceride.

### Association of normal LDL-c levels with prevalence rates of NAFLD

As shown in Table 1, the prevalence of NAFLD from Q1 to Q4 was 19.34%, 25.86%, 35.65% and 42.08% respectively. To further understand the relationship between LDL-c level and the prevalence of NAFLD, the OR for NAFLD were calculated after adjusting for confounding variables. Using Q1 as a reference, the OR for NAFLD was 1.45 (95% CI 1.31-1.61), 2.31 (95% CI 2.11-2.53), 2.31 (95% CI 2.11, 2.53) for Q2, Q3, and Q4, respectively in model 1. Adjustment for age, sex, BMI (model 2) substantially attenuated the magnitude of the OR when comparing Q4 with Q1. In the fully adjusted model (model 3), the relationship between LDL-c and NAFLD remained statistically significant in Q2, Q3 and Q4 with OR of 1.31 (95% CI 1.14-1.54), 1.73 (95% CI 1.46-2.04) and 1.82 (95% CI 1.49-2.23), respectively (Table 3). These results suggest that patients with higher LDL-c levels are more likely to develop NAFLD than subjects with lower LDL-c levels.

**Table 3:** Adjusted Odds Ratio or Hazard ratio (95% Confidence Interval) for Nonalcoholic Fatty Liver Disease

Quartiles of LDL-C	NAFLD Case	Model 1	Model 2	Model 3
Cross-section population				
Q1	953 (4926)	1.00 (1.00, 1.00)	1.00 (1.00, 1.00)	1.00 (1.00, 1.00)
Q2	872 (3372)	1.45 (1.31, 1.61)	1.25 (1.10, 1.42)	1.31 (1.14, 1.54)
Q3	1815 (5091)	2.31 (2.11, 2.53)	1.63 (1.46, 1.82)	1.73 (1.46, 2.04)
Q4	2049 (4869)	3.02 (2.77, 3.32)	1.84 (1.65, 2.05)	1.82 (1.49, 2.23)
*P* value		<0.001	<0.001	<0.001
Longitudinal population				
Q1	837 (5739)	1.00 (1.00, 1.00)	1.00 (1.00, 1.00)	1.00 (1.00, 1.00)
Q2	945 (5087)	1.23 (1.12, 1.35)	1.19 (1.08, 1.31)	1.18 (1.05, 1.33)
Q3	1183 (5116)	1.57 (1.44, 1.72)	1.28 (1.17, 1.40)	1.19 (1.04, 1.38)
Q4	1288 (4491)	2.02 (1.86, 2.21)	1.57 (1.43, 1.72)	1.46 (1.27, 1.70)
*P* value		<0.001	<0.001	<0.001

Model 1 is univariate analysis. Model 2 is adjusted for sex, age, body mass index. Model 3 is adjusted for sex, age, body mass index, systolic blood pressure, fasting plasma glucose, albumin, alanine aminotransferase, aspartate aminotransferase, blood urea nitrogen, creatinine, serum uric acid, total cholesterol, triglyceride, high-density lipoprotein cholesterol and uric acid.

**Figure 2:**
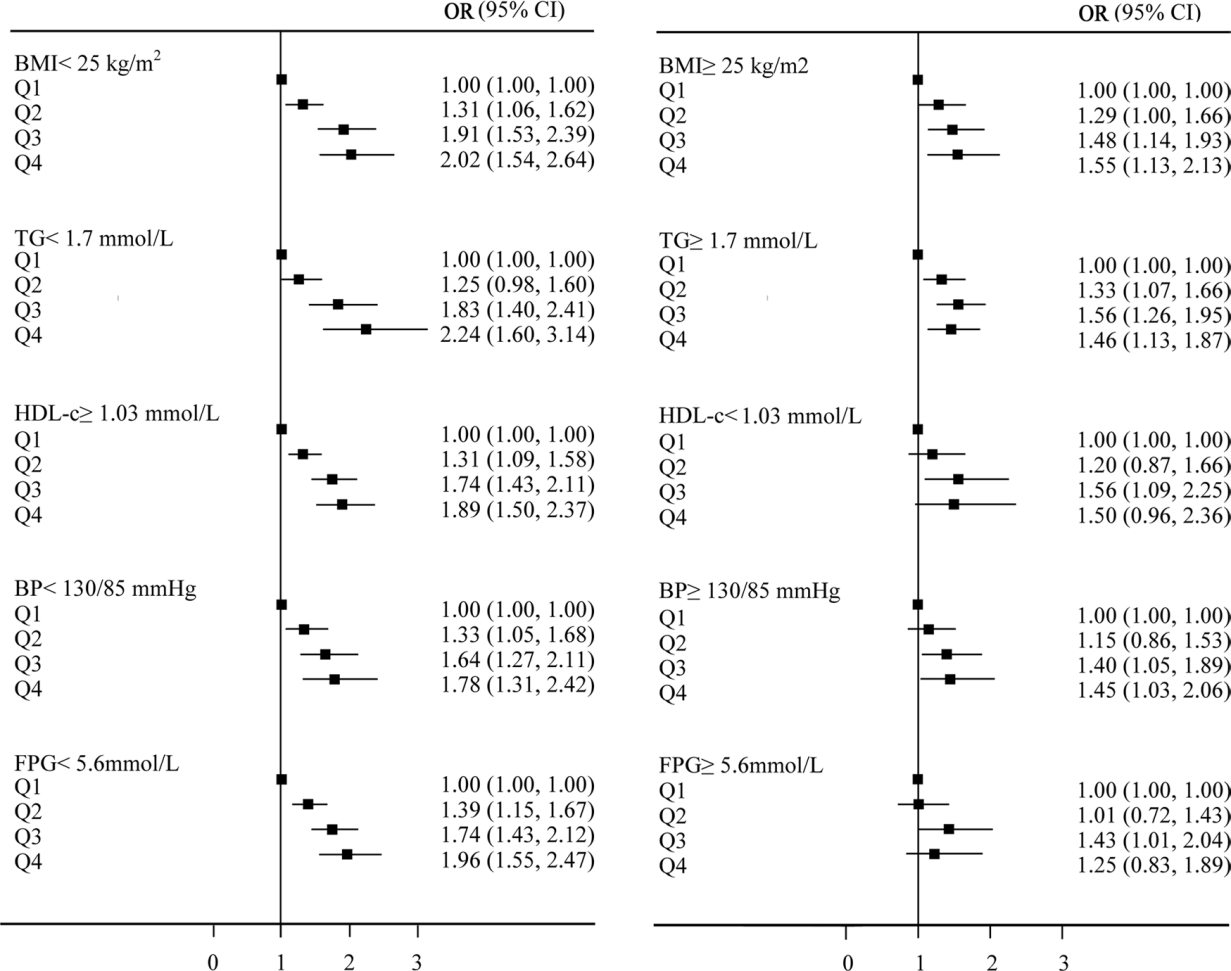
Forest plots of odds ratios (OR) (95% confidence interval [CI]) for quartiles of LDL-c in the cross-sectional population Confounding variables contained age, sex, body mass index, systolic blood pressure, fasting plasmaglucose, albumin, alanine aminotransferase, aspartate aminotransferase, blood urea nitrogen, creatinine, total cholesterol, triglyceride, high-density lipoprotein cholesterol, and uric acid. Increasing trends of OR for NAFLD with the increases in normal LDL-c levels are shown. Q1: ≤ 2.00 mmol/L, Q2: 2.10-2.35 mmol/L, Q3: 2.36-2.68 mmol/L and Q4: 2.69-3.12 mmol/L.

Figure 2 shows forest plots of OR for quartiles of LDL-c in the cross-sectional population. A stratified analysis for risk factors of metabolic syndrome showed a successive increase in OR from Q1 to Q4. The strongest link between increasing levels of LDL-c and the prevalence of NAFLD was observed in subjects with TC < 1.7 mmol/L (OR _Q4 VS. Q1_ was 2.24, 95% CI 1.60-3.14). The weakest link was observed in subjects with FPG ≥ 5.6 mmol/L (OR _Q4 VS. Q1_ was 1.25 95% CI 0.83-1.89).

### Higher LDL-c within normal range predicts increase risk of NAFLD

**Figure 3:**
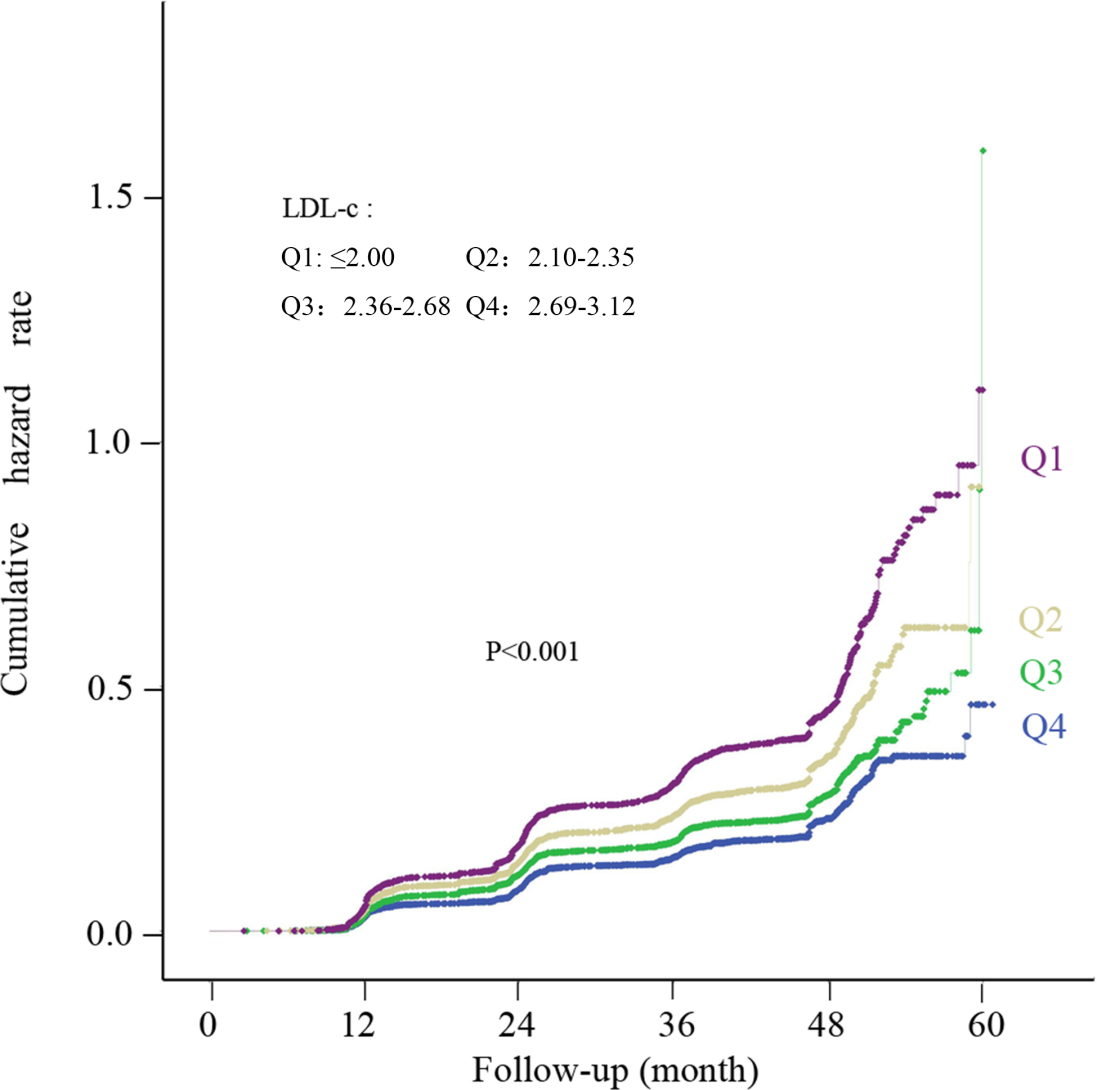
Kaplan-Meier curves reflecting cumulative incidence rate of NAFLD in the longitudinal population according to quartiles of normal LDL-c level Subjects with higher LDL-c level within the normal range had an increased cumulative incidence rate of NAFLD. *P* value for trend is computed from Cox analysis.

**Figure 4:**
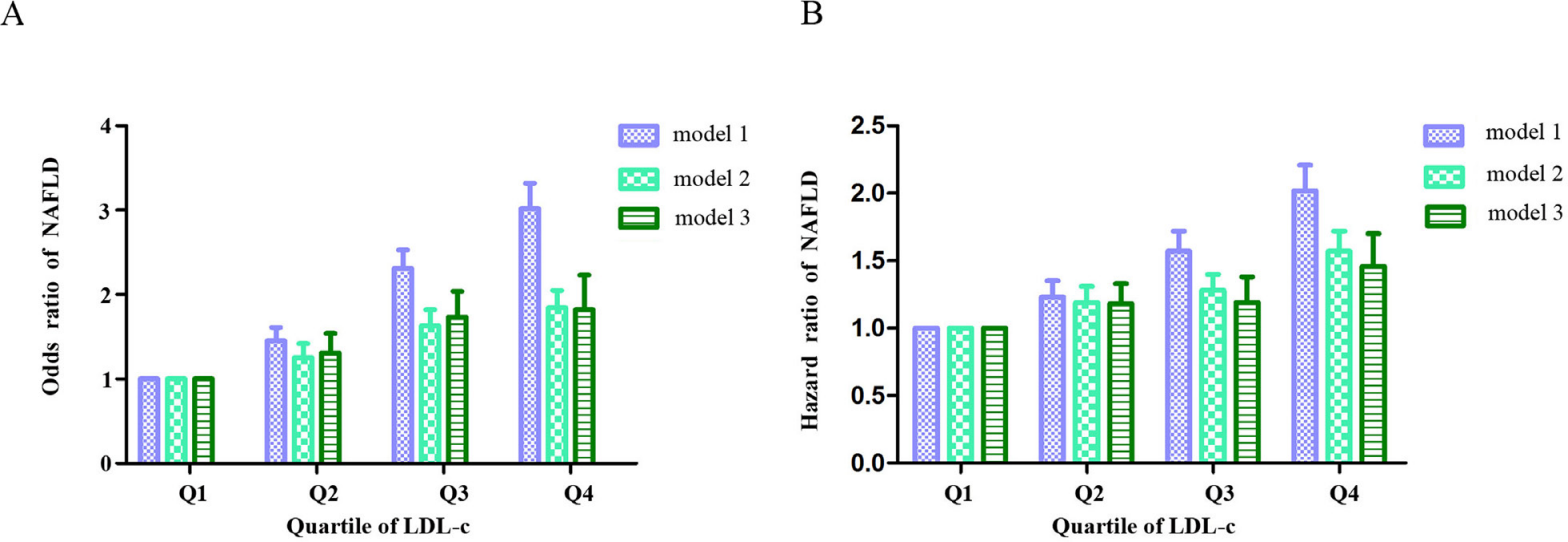
Unadjusted and adjusted odds ratios (OR) and hazard ratios (HR) for NAFLD **A.** and **B.** showed the OR and HR of LDL-c in the cross-sectional population and longitudinal population, respectively. Model 1 is a univariate analysis. Model 2 is adjusted for sex, age, body mass index. Model 3 is adjusted for sex, age, body mass index, systolic blood pressure, fasting plasma glucose, albumin, alanine aminotransferase, aspartate aminotransferase, blood urea nitrogen, creatinine, serum uric acid, total cholesterol, triglyceride, high-density lipoprotein cholesterol and uric acid. Q1: ≤ 2.00 mmol/L, Q2: 2.10-2.35 mmol/L, Q3: 2.36-2.68 mmol/L and Q4: 2.69-3.12 mmol/L.

To verify whether an increased level of LDL-c with the normal range may play a causal role in the development of NAFLD, a longitudinal population was included. 20433 subjects finally completed follow-up data, which 4253 subjects had developed into NAFLD. In unadjusted model 1, the HR for NAFLD was 1.23 (95% CI 1.12-1.35), 1.57 (95% CI 1.44-1.72), 2.02 (95% CI 1.86-2.21) for Q2, Q3, and Q4, respectively. Adjusting for fully adjusted model (model 3), the relationship between LDL-c and NAFLD remained significant in Q2, Q3 and Q4 with HR of 1.18 (95% CI 1.05-1.33), 1.19 (95% CI 1.04-1.38) and 1.46 (95% CI 1.27-1.70), respectively (Table 3). Figure 3 shows the cumulative HR of NAFLD in groups of LDL-c by Kaplan-Meier analysis. Figure 4A and 4B shows the unadjusted and adjusted OR and HR of normal LDL-c levels for NAFLD cross-sectional population and longitudinal population, respectively. These results indicated that normal LDL-c level may be an important factor that predicts the development of NAFLD and the risk may increase with an increased level of LDL-c.

## DISCUSSION

Dyslipidemia in patients with NAFLD is characterized by increased levels of serum triglycerides and decreased levels of HDL-c [6,15]. Previous studies have demonstrated that patients with NAFLD have significantly increased levels of oxidized LDL-c [16–17], LDL-migration index [18], which are both highly atherogenic. There are important differences in the LDL-c and HDL-c subfractions in patients with NAFLD [17, 19–20], however, there has been no examination of the relationship between LDL-c within the normal range and the prevalence of NAFLD. To our knowledge, our study is the first and largest analysis specifically led to evaluate the association between normal LDL-c range and NAFLD risk in a nationally representative sample. We observed a significant association between LDL-c level and prevalence of NAFLD in the cross-sectional population. Firstly, the prevalence of NAFLD gradually increased as the LDL-c level increased. Secondly, the stratified analysis demonstrated that the relationship between the LDL-c and metabolic syndrome components indirectly predicted the correlation between NAFLD and LDL-c. Thirdly, logistic regression analysis further showed the elevated LDL-c level in normal range importantly contributed to the risk for NAFLD. Our results are in agreement with prior studies [12,20].

Furthermore, a prospective longitudinal population was performed to verify that the elevation of LDL-c level within the normal range appears to make a significant contribution to an increased risk of developing NAFLD. Fully adjusted for confounding variables, the relationship between LDL-c and NAFLD remained significant in Q4 with HR of 1.46 (95% CI 1.27-1.70). Thus, higher normal LDL-c level appears to increase the incidence risk of NAFLD.

LDL-c consists of large quantities of cholesteryl ester, whose production is dependent on dissociation from the exchangeable apoliproproteins of VLDL by lipoprotein lipase [21]. One possible explanation for the relationship between LDL-c level and NAFLD is insulin resistance. Insulin resistance can mechanistically explain many of the key alterations observed in lipoprotein metabolism, which leads to increasing lipolysis within white adipose tissue and concomitant increased delivery of free fatty acids to the liver and increased expression of hepatic fatty acid transport proteins [22]. In the setting of insulin resistance, hepatic lipase can be upregulated and multiple metabolic abnormalities may conspire to increase the secretion of VLDL particles [23]. Therefore, all these factors are likely to contribute to increasing the proportion of small dense LDL particles in this metabolic state. In a 32-month follow-up study, we have demonstrated that there was a significant co-existence of high levels of oxidized LDL-c, oxidized LDL-c/HDL-c, and insulin resistance [24]. As NAFLD is a condition closely related to insulin resistance, it may partially explain why elevation of LDL-c appears to significantly increase the risk of NAFLD.

However, an association between LDL-c levels and NAFLD was still observed after adjusting for features of metabolic syndrome and other known confounding variables. The MESA study demonstrated that primary lipoprotein abnormalities resulting in hepatic triglyceride over production or impaired secretion, independent of insulin resistance may be responsible for NAFLD [12]. Liver fat content is directly associated with VLDL-apoB100 concentrations and a defect in postprandial apolipoprotien B secretion, leading to triglyceride accumulation has been demonstrated in hepatic steatohepatitis [25–26]. An overproduction of apolipoprotein B100 containing particles (large VLDL, VLDL, intermediate-density lipoprotein) observed are released by the liver and converted to cholesterol rich LDL particles via lipoprotein lipase [12]. However, further studies investigating the molecular and cellular mechanisms of LDL-c in NAFLD are required.

Low-density lipoprotein receptor-related protein 6 (LRP6) is a member of the LDL receptor family and is essential for normal LDL clearance. It has a unique structure and plays a pivotal role in metabolic regulation [27]. Investigation of the relationship between LRP6 and lipid synthesis in the liver was accomplished using a human LRP6 mutation-carrying mouse model [28]. The mutant mice had elevated TG and cholesterol synthesis, resulting in lipid accumulation in their livers by activation of the rapamycin (mTOR) pathway [29]. Furthermore, patients carrying an LRP6 mutation exhibit elevated levels of LDL-c, TC, and fasting glucose, which constitute the risk factors of the diseases including hypertriglyceridemia [30], hypercholesterolemia [31], atherosclerosis [32] and NAFLD [28]. These findings have greatly advanced the understanding of disease pathogenesis.

Our study may have some limitations and merit comment. The main limitation is lack of anthropometric parameters regarding central obesity, lifestyle, and dietary factors, which may be helpful to better understand the relationship between NAFLD and LDL-c levels. Secondly, LDL subclasses and LDL-migration index in different stages of NAFLD should be considered, since it is important and meaningful to identify the differences between subjects with SS and NASH. Thirdly, although the use of liver biopsy is the gold standard for assessing NAFLD, the ultrasonography is widely used in epidemiological surveys of NAFLD because of its safety, economical and practical utility.

In conclusion, we have demonstrated that increased levels of LDL-c within the normal range have independently relationship with an elevated risk of NAFLD in the both a cross-sectional and longitudinal population. The LDL-c levels within the normal range appear to play a significant role on the prevalence and incidence of NAFLD. Therefore, we propose that LDL-c evaluation and control should be an integral component of clinical management of the general population. Surveillance and treatment of dyslipidemia is therefore paramount for the prevention and treatment of NAFLD and cardiovascular disease.

## MATERIALS AND METHODS

### Study design

The cross-sectional population consisted of 27374 individuals who underwent a health examination in the First Affiliated Hospital of Wenzhou Medical University from January 2010 to December 2010. The longitudinal population was based on a prospective study and conducted from 33153 initially NALFD-free individuals who underwent an annual health examination in Wenzhou Medical Center of Wenzhou People's Hospital. The study period was initiated in January 2010 and concluded in December 2014.

The exclusion criteria were as follows: alcohol abuse; those taking antihypertensive agents, antidiabetic agents, lipid-lowing agents; other known causes of chronic liver disease; LDL-c > 3.12mmol/L and subjects whose data were missing or who were lost to follow-up.

Verbal informed consent was obtained from each subject before their participation in the study. The personal information of subjects was erased and replaced by the health examination number. The research protocol of the study was approved by the ethics committee of the First Affiliated Hospital of Wenzhou Medical University and Wenzhou People's Hospital, respectively.

### Diagnostic criteria

A diagnosis of NAFLD was made in reference to Guidelines for the assessment and management of NAFLD in Asia-Pacific region [33]. In general, NAFLD can be diagnosed when imaging tests indicate hepatic steatosis, excluding alcohol abuse and specific diseases that could lead to steatosis. Hepatic steatosis was defined by the presence of at least 2 of 3 abnormal findings on abdominal ultrasonography: diffusely increased echogenicity (“bright”) liver with liver echogenicity greater than kidney or spleen, vascular blurring, or deep attenuation of ultrasound signal. The ultrasound was assessed by 2 experienced imaging specialists who were blinded to the examinee history and the study during the ultrasonic examination. A third imaging specialist was invited if the diagnoses made by the 2 imaging specialists were not in agreement or inconclusive. Metabolic syndrome represents a cluster of physiological and anthropometric abnormalities, requiring ≥ 3 of the following 5 factors: (1) waist circumference ≥ 90 cm in men, ≥ 80 cm in women (2) serum triglyceride ≥1.7 mmol/L (3) high density lipoprotein cholesterol <1.03 mmol/L in men, <1.29 mmol/L in women (4) systolic blood pressure ≥ 130 mmHg or diastolic blood pressure ≥85 mmHg (5) fasting glucose ≥5.6 mmol/L.

### Data collection

Clinical examination and data recording were conducted in the morning after an overnight fast and subjects were also instructed to refrain from exercise during the day before their examination. Medical history and a health habit inventory were taken by a physician.

Standing height and body weight were measured without shoes or thick clothing. Body mass index (BMI, kg/m^2^), used as an index of body fat, was calculated as weight in kilograms divided by height in meters squared. Blood pressure, including systolic blood pressure (SBP) and diastolic blood pressure (DBP), was measured using an automated sphygmomanometer with the subject in a quite environment and in a sitting position.

Fasting blood samples were collected from each subject in an antecubital vein and were used for the analysis of biochemical measurements serum samples without frozen. The experimental procedures were consistent throughout the study period and the laboratories were both certified according to International Oragnization Standardization. The biochemical measurements included albumin (ALB), alanine aminostransferase (ALT), aspartate aminotranferase (AST), fasting plasma glucose (FPG), blood urea nitrogen (BUN), creatinine (Cr), uric acid (UA), total cholesterol (TC), triglyceride (TG), HDL-c, and LDL-c. All values were measured by an automated analyzer (Abbott AxSYM) using standard methods.

### Statistical analysis

In order to derive a deeper understanding of the relationship between normal range of LDL-c levels and the prevalence of NAFLD, all subjects were classified into 4 groups by quartiles statistically. Quartiles in the cross-sectional population were categorized separately as follows: Q1: ≤ 2.0 mmol/L, Q2: 2.1-2.35 mmol/L, Q3: 2.36-2.68 mmol/L, Q4: 2.69-3.12 mmol/L. The grouping in the longitudinal population had the same LDL-c range as the cross-sectional population.

In the cross-sectional population, the OR and 95% confidence intervals (CIs) for NAFLD were calculated after adjusting for known confounding variables across each quartile of LDL-c concentration using multivariate logistic regression analysis. HR based on Cox's proportional hazards regression were determined in the longitudinal population analysis. Kaplan-Meier analysis was applied to calculate the cumulative hazard of NAFLD during the follow-up. Multivariable models included sex, age, BMI, FPG, ALB, ALT, AST, BUN, Cr, SUA, TC, TG, HDL-c, SBP and UA.

Continuous variables were summarized as mean ± standard deviation (SD), and categorical variables were displayed as counts or percentages (%). The characteristics of the study population according to LDL-c quartiles were compared using a one-way analysis of variance (ANOVA) for continuous variables and χ^2^-test for categorical variables. All *P*-values are 2-sided and a *P* value of <0.05 was considered statistically significant. Analyses were performed in SPSS version 18.0 (SPSS, Chicago, IL).

## Abbreviations

ALB: albumin
ALT: alanine aminotransferase
AST: aspartate aminotransferase
BMI: body mass index
BUN: blood urea nitrogen
CIs: confidence intervals
HR: hazard ratios
Cr: creatinine
DBP: diastolic blood pressure
FPG: fasting plasma glucose
HDL: high-density lipoprotein
HDL-c: high-density lipoprotein cholesterol
LDL: low-density lipoprotein
LDL-c: low-density lipoprotein cholesterol
LRP6: Low-density lipoprotein receptor-related protein 6
VLDL: very low density lipoprotein
SD: standard deviation
NAFLD: nonalcoholic fatty liver disease
OR: odds ratios
UA: uric acid
SBP: systolic blood pressure
TC: total cholesterol
TG: triglyceride
NASH: nonalcoholic steatohepatitis
CVD: cardiovascular disease
